# Ecotoxicity and genetic toxicity data from a pulp mill bleaching effluent treated with anaerobic digestion and advanced oxidation process (AOP)

**DOI:** 10.1016/j.dib.2020.105141

**Published:** 2020-01-16

**Authors:** Tatiana R. Chaparro, Juan Gabriel Rueda-Bayona

**Affiliations:** a Military University, Civil Engineering Department, Environmental Sanitation Laboratory, Water and Energy (AyE) Research Group, Bogotá, Carrera 11 No.101- 80, Colombia; b Military University, Civil Engineering Department, Civil Engineering, Water and Energy (AyE) Research Group, Bogotá, Carrera 11 No.101- 80, Colombia

**Keywords:** *Allium cepa* L, Anaerobic treatment, *Ceriodaphnia* spp, *Daphnia* spp, Ozone, Ozone/UV

## Abstract

Wastewater treatment contributes to environmental sustainable development indicators such as clean water and sanitation, then, is imperative to improve the mechanisms and process of contaminant removals. The sewage and industrial effluents are the major contributors of pollutants in land and water discharges, and are necessary to enrich the available data for having reference parameters for plant designing and optimization. The physical andchemical assays alone could not be considered sufficient to assess properly the plant performance because complex mixtures demand ecological and biological parameters for a holistic evaluation. Hence, the ecotoxicity and the genetic toxicity measurement become an important tool to complement the conventional water quality parameters, but these parameters are not widely reported in the open access literature. Despite of several studies showed ecotoxicity and the genetic toxicity data, these could be considered not sufficient because the resulted information is derived from single compounds. Considering the scarce data mentioned above this article presents data on the genetic an ecological toxicity of an anaerobic effluent post-treated with ozone and ozone/UV generated by Chaparro et al. [1] and Chaparro and Pires [2].

**Table:** Specifications Table

Subject area	Environmental science
More specified Subject area	Health, Toxicology and Mutagenesis
Type of data	Table, Excel Spreadsheets and images.
How data were acquired	All bioassays used the anaerobic effluent after ozone and ozone/UV treatment. Acute and Chronic toxicity of *Daphnia* spp and *Ceriodaphnia* spp were measured in EC_50_ and ICP_25_ respectively. The genetic toxicity was evaluated with the meristematic region of the *Alllium cepa* L roots.
Data format	Raw and calculated
Experimental factors	Acute and Chronic toxicity were expressed with acute and chronical units calculated as TU_C_: 100/EC_50_, TU_A_: 100/ICP_25_. The genetic toxicity was evaluated through the mitotic, cytotoxic and mutagenic index calculated with the number of cells observed for each case.
Experimental features	The samples were taken at regular intervals during the observation period of the anaerobic bioreactor, operating in continuous and ozone and ozone/UV in batch mode.
Data source location	Sao Paulo-SP (Brazil)
Data accessibility	With this article and doi: https://doi.org/10.17632/nf7v867ppm.1, https://doi.org/10.17632/fjn33cfg82.1

**Table:** 

**Value of the Data** • The data presented in this article can be used as complementary tool to evaluate the performance of the combination of anaerobic digestion with advanced oxidation process treating complex mixtures. • The data obtained from bioassays in particular from the higher plants as the *Allium cepa* L. *cells* provides relevant information about the genetic effects of an industrial wastewater. • This data contains useful information for optimizing biological processes and treatment of industrial wastewater with non-conventional pollutants. • This data contributes to the construction of new industrial discharge limits considering bioassays as wastewater quality indicators. • The data indicates that in mixture complex such as Pulp mill effluents is necessary implementing bioassays with different trophic levels to evaluate the toxic effects.

## 1 Data

**Fig. 1:**
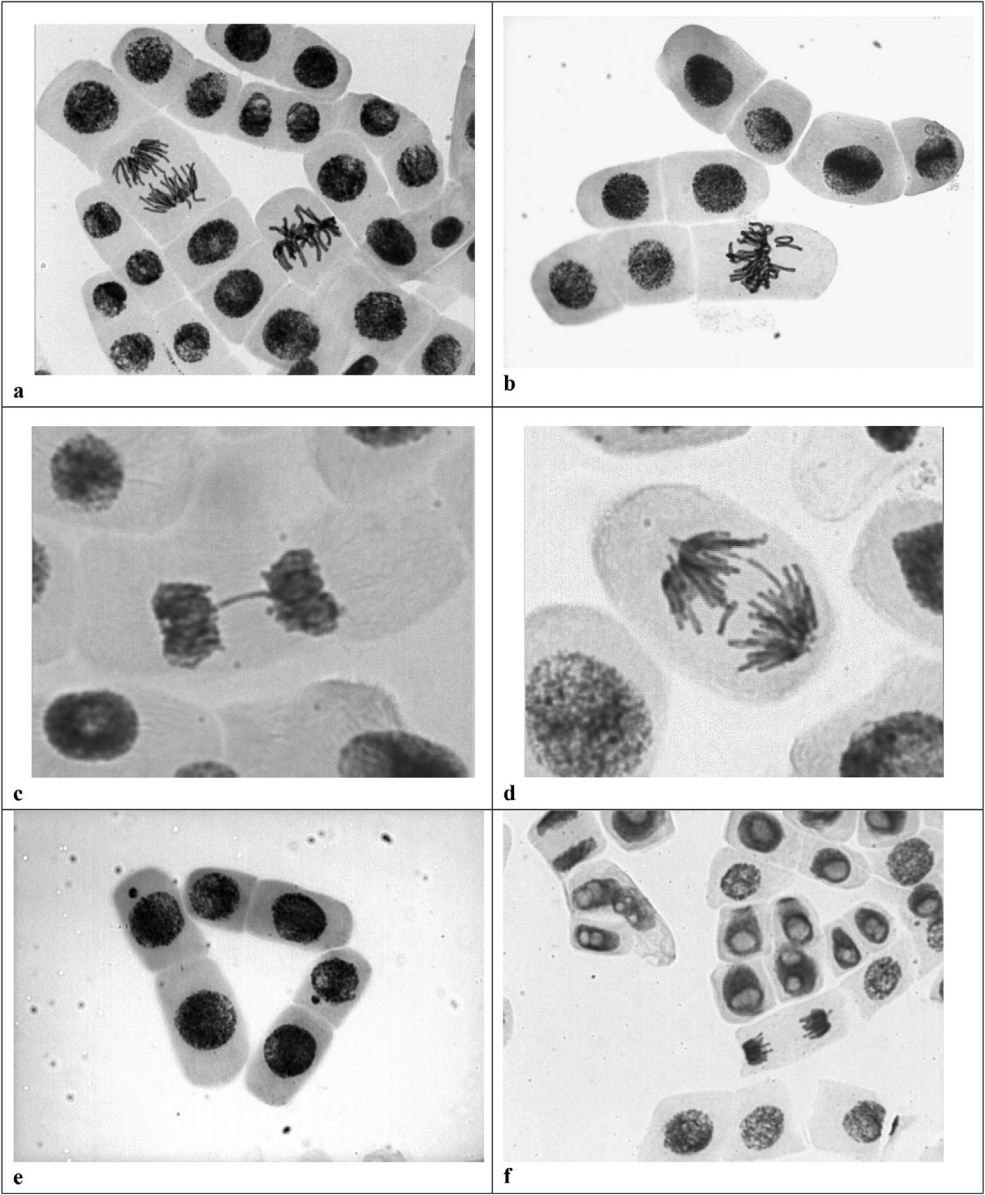
Cells in root tips of *Allium cepa* exposed with bleaching effluents and the HAIB reactor. (a) normal cellular division, anaphase, metaphase, interphase. (b) Disturbed metaphase with break. (c) Telophase with bridge. (d) Anaphase with loss and bridge. (e) Micronucleus. (f) Binucleus cells and multiple nuclear lesions.

**Fig. 2:**
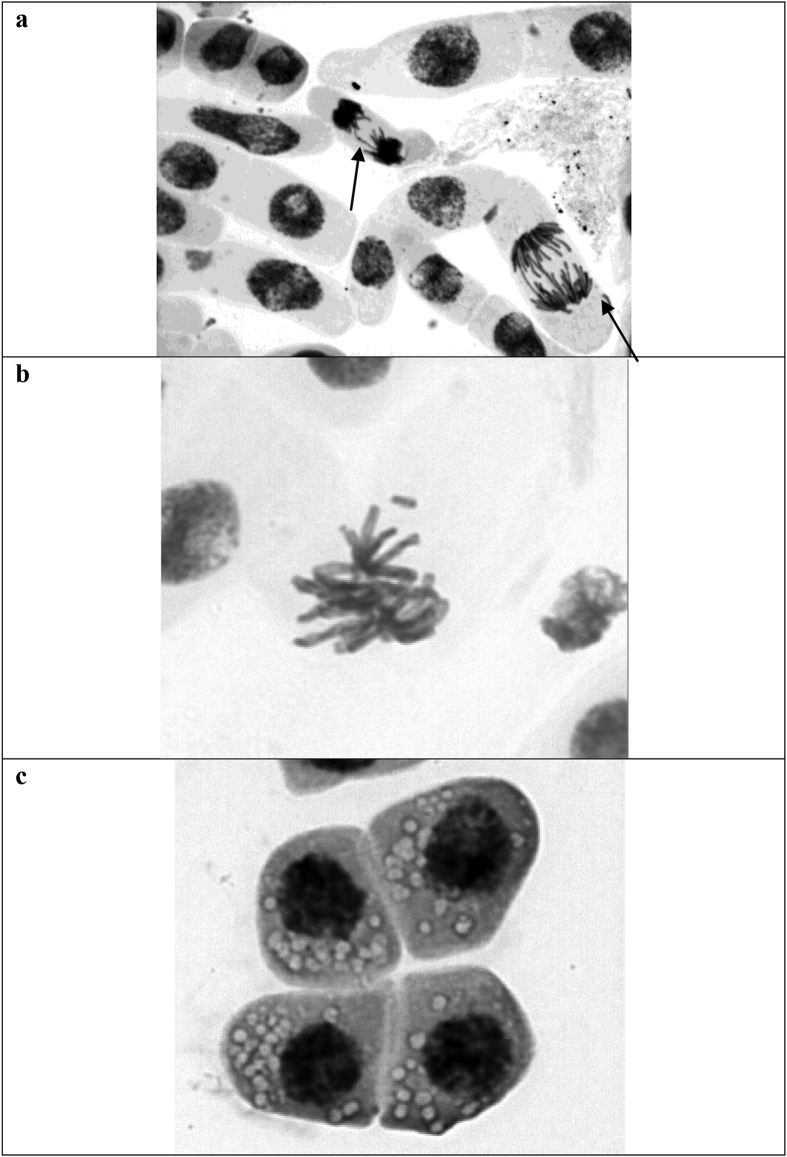
Cells in root tips of *Allium cepa* exposed with ozone and ozone/UV (a) Telophase with bridge and anaphase with bridge and break. b) Metaphase with loss. C) Vacuolized cell.

The Table 1 resumes the main values derived from the ecotoxicology assays and the Table 2 presents the main characteristic of these measurements. The raw data of the acute and chronic toxicity are shown in: Table 3, Table 4, Table 5, Table 6, Table 7, Table 8, Table 9, Table 10, Table 11, Table 12, Table 13, Table 14, Table 15, Table 16, Table 17, Table 18, Table 19. The Genetic toxicity was evaluated considering the results of the Chromosome aberrations index (CA), variation of the mitotic index (IM) and mutagenic effects as number of micronucleus (MN). The data is gathered in a Excel sheet file available in the Mendeley data website which can be found with the title “Ecotoxicity and genetic toxicity data (Allium cepa) from a bleaching wastewater treated on an anaerobic process and “Ecotoxicity and genetic toxicity data of an anaerobic effluent pos-treated with ozone and ozone/UV” respectively. Finally, Fig. 1 and Fig. 2 show images of the main genetic effects observed after the treatments.

**Table 1:** Main values of the Acute and Chronic toxicity.

Treatment	EC_50_	EC_50_	ICp_(25)_	ICp_(25)_
Raw WW	5.47 5.36–5.59	7.42 7.24–7.62	4.93	5.52 Not available
Anaerobic _effluent_	59.95 52.71–68.20	67.6 61–74	3.82 3.42–4.54	27.08 15.45–30.59
Ozone _effluent_	65.98 60.12–72.41	79.58 76.47–82.84	16.22 15.50–16.65	18.31 17.19–18.68
O_3_/UV _effluent_	64.04 57.61–71.20	77.97 73.97–82.39	16.41 15.99–17.03	13.79 13.22–14.61

**Table 2:** Main requirements for the maintenance of the cultures during the ecotoxicity assessment.

Requirements	*Daphnia similis*	*Ceriodaphnia dúbia/silvestrii*
Assay	Static	semi-static
Duration	48 hours	7 days
Temperature	20 ± 5 °C	
Photoperiod/light intensity.	16h light: 8h dark 500–1000 lux	
Volume of samples	10 mL	15 mL
Minimum number of dilutions with replicates	Five more controls	
Number of replicates per dilution.	4	10
Feeding	Not	Yes
Water of dilution	Reconstituted water tank	
Test organism age	6h–24h	
Number of organisms per replicate	5	1
Renewal of the test solution	Not	Every 2 days
Evaluation criteria	Mortality/Immobility	Reproduction/Survival
Test Acceptance Criteria	>90% survival of organisms in the control	>80% survival and ≥15 neonates/female control.

**Table 3:** Acute toxicity of industrial bleaching effluent (test 1).

Sample	Concentration (%)	number exposed	Mortalities^a^
1	5	20	0
2	5.8	20	18
3	6.9	20	20
4	8.8	20	20
5	10	20	20

a Statistical validation. Spearman-Karber trim = 0; Spearman-Karber estimates = eC50–5.472590; lower confidence interval (95%) = 5.36; upper confidence interval (95%) = 5.59.

**Table 4:** Acute toxicity of industrial bleaching effluent (test 2).

Sample	Concentration (%)	number exposed	Mortalities^a^
1	5.7	20	0
2	6.9	20	2
3	8.3	20	20
4	10	20	20

a Statistical validation. Spearman-Karber trim = 0; Spearman-Karber estimates = eC50–7.4268293; lower confidence interval (95%) = 7.24; upper confidence interval (95%) = 7.62.

**Table 5:** Chronic Toxicity of industrial bleaching effluent (test 1).

Sample.	Number of Replicates	Concentration (%)	Mean response	Standard deviation	Pooled^a^ mean response
1	10	0	13.5	3.064	13.5
2	10	3.8	12.3	6.977	12.3
3	10	4.5	11.6	7.106	11.6
4	10	5.4	10.3	6.516	10.3
5	10	6.5	8.7	4.498	8.7

a Statistical validation. Linear Interpolation Estimate = 5.5203; Entered P Value = 25; number of resampling = 80; Resamples Generated = 71, those resamples not used had estimates above the highest concentration; The Bootstrap Estimates Mean = 4.9619; standard deviation = 0.9109; No Confidence Limits can be produced since the number of resamples generated is not a multiple of 40; resampling time in Seconds = 0.00; random Seed: 435879320.

**Table 6:** Chronic Toxicity of industrial bleaching effluent (test 2).

Sample.	Number of Replicates	Concentration (%)	Mean response	Standard deviation	Pooled^a^ mean response
1	10	0	10.9	3.573	10.9
2	10	2.2	10.3	2.058	10.3
3	10	3.3	10.2	1.229	10.2
4	10	5	8.1	1.912	8.1

a Statistical validation. Linear Interpolation Estimate = 4.9393; Entered P Value = 25; number of resampling = 80; Resamples Generated = 36, those resamples not used had estimates above the highest concentration; The Bootstrap Estimates Mean = 4.9619; standard deviation = 0.9109; No Confidence Limits can be produced since the number of resamples generated is not a multiple of 40; resampling time in Seconds = 0.00; random Seed: 314160245.

**Table 7:** Acute toxicity of HAIB reactor effluent (test 1).

Sample	Concentration (%)	number exposed	Mortalities (*Daphnia similis*)^a^
1	6.25	20	0
2	12.50	20	0
3	25.00	20	0
4	50.00	21	5
5	100.00	20	20

a Statistical validation. Spearman-Karber trim = 0; Spearman-Karber estimates = eC50–59.9530334; lower confidence interval (95%) = 52.71; upper confidence interval (95%) = 68.20.

**Table 8:** Acute toxicity of HAIB reactor effluent (test 2).

Sample	Concentration (%)	number exposed	Mortalities (*Daphnia similis*)^a^
1	20	20	0
2	30	20	0
3	44	20	2
4	66	20	7
5	100	20	20

a Statistical validation. Spearman-Karber trim = 0; Spearman-Karber estimates = eC50–67.6480331; lower confidence interval (95%) = 61.07; upper confidence interval (95%) = 74.93.

**Table 9:** Acute toxicity of HAIB reactor effluent (test 3).

Sample	Concentration (%)	number exposed	Mortalities (*Daphnia similis*)^a^
1	6.25	20	0
2	12.50	20	0
3	25.00	20	0
4	50.00	20	1
5	100.00	20	20

a Statistical validation. Spearman-Karber trim = 0; Spearman-Karber estimates = eC50–68.3019943; lower confidence interval (95%) = 63.84; upper confidence interval (95%) = 73.08.

**Table 10:** Chronic Toxicity of HAIB reactor effluent (test 3).

Sample.	Number of Replicates	Concentration (%)	Mean response	Standard deviation	Pooled^a^ mean response
1	9	0	7.667	1.323	7.667
2	10	12.5	1.4	1.35	1.4
3	10	25	0	0	0

a Statistical validation. Linear Interpolation Estimate = 3.8231; Entered P Value = 25; number of resampling = 80; Resamples Generated = 80, those resamples not used had estimates above the highest concentration; The Bootstrap Estimates Mean = 3.8683; standard deviation = 0.2846; resampling time in Seconds = 0.00; random Seed: -181298520.

**Table 11:** Chronic Toxicity of HAIB reactor effluent (test 3).

Sample.	Number of Replicates	Concentration (%)	Mean response	Standard deviation	Pooled^a^ mean response
1	10	0	11.1	6.999	12.824
2	7	11.85	15.286	5.345	12.824
3	10	17.7	9.8	5.673	9.9
4	10	26.6	10	6.146	9.9
5	10	40	2.1	2.807	2.1

a Statistical validation. Linear Interpolation Estimate = 27.0851; Entered P Value = 25; number of resampling = 80; Resamples Generated = 80; the Bootstrap Estimates Mean = 23.1732; standard deviation = 5.4741; resampling time in Seconds = 0.00; random Seed: -1470801740. lower confidence interval (95%) = 15.4527; upper confidence interval (95%) = 30.5956.

**Table 12:** Acute toxicity of industrial bleaching effluent treated with ozone/UV (test 1).

Sample	Concentration (%)	number exposed	Mortalities^a^
1	6.25	20	0
2	12.50	20	0
3	25.00	20	0
4	50.00	21	3
5	100.00	20	20

a Statistical validation. Spearman-Karber trim = 0; Spearman-Karber estimates = eC50 64.04; lower confidence interval (95%) = 57.61; upper confidence interval (95%) = 71.20.

**Table 13:** Acute toxicity of industrial bleaching effluent treated with ozone/UV (test 2).

Sample	Concentration (%)	number exposed	Mortalities^a^
1	20.00	20	0
2	30.00	20	0
3	44.00	19	0
4	66.00	20	2
5	100.00	20	20

a Statistical validation. Spearman-Karber trim = 0; Spearman-Karber estimates = eC50 77.97; lower confidence interval (95%) = 73.79; upper confidence interval (95%) = 82.39.

**Table 14:** Acute toxicity of industrial bleaching effluent treated with ozone (test 1).

Sample	Concentration (%)	number exposed	Mortalities^a^
1	6.25	20	0
2	12.50	20	0
3	25.00	20	0
4	50.00	20	2
5	100.00	20	20

a Statistical validation. Spearman-Karber trim = 0; Spearman-Karber estimates = eC50 65.97; lower confidence interval (95%) = 60.12; upper confidence interval (95%) = 72.41.

**Table 15:** Acute toxicity of industrial bleaching effluent treated with ozone (test 2).

Sample	Concentration (%)	number exposed	Mortalities^a^
1	20.00	19	0
2	30.00	20	0
3	44.00	18	0
4	66.00	20	1
5	100.00	20	20

a Statistical validation. Spearman-Karber trim = 0; Spearman-Karber estimates = eC50 79.59; lower confidence interval (95%) = 76.47; upper confidence interval (95%) = 82.84.

**Table 16:** Chronic Toxicity of industrial bleaching effluent treated with ozone (test 1).

Sample.	Number of Replicates	Concentration (%)	Mean response	Standard deviation	Pooled^a^ mean response
1	9	0	7.67	1.323	8.68
2	10	12.50	9.60	4.40	8.68
3	10	25.00	1.40	1.51	1.40

a Statistical validation. Linear Interpolation Estimate = 16.2256; Entered P Value = 25; number of resampling = 80; Resamples Generated = 80; The Bootstrap Estimates Mean = 16.1503; standard deviation = 0.3784; lower confidence interval (95%) = 15.5014; upper confidence interval (95%) = 16.6576. resampling time in Seconds = 0.00; random Seed: -347644830.

**Table 17:** Chronic Toxicity of industrial bleaching effluent treated with ozone (test 2).

Sample.	Number of Replicates	Concentration (%)	Mean response	Standard deviation	Pooled^a^ mean response
1	10	0	12.6	2.989	13
2	10	15.8	13.4	4.624	13
3	10	23.7	2.8	2.348	2.8
4	10	35.55	1.1	0.876	1.1

a Statistical validation. Linear Interpolation Estimate = 18.3172; Entered P Value = 25; number of resampling = 80; Resamples Generated = 80; The Bootstrap Estimates Mean = 18.1233; standard deviation = 0.7975; lower confidence interval (95%) = 17.1929; upper confidence interval (95%) = 18.6884. resampling time in Seconds = 0.00; random Seed: -27183750.

**Table 18:** Chronic Toxicity of industrial bleaching effluent treated with ozone/UV (test 1).

Sample.	Number of Replicates	Concentration (%)	Mean response	Standard deviation	Pooled^a^ mean response
1	9	0	7.667	1.323	8.421
2	10	12.5	9.1	2.644	8.421
3	10	25	1.7	1.829	1.7

a Statistical validation. Linear Interpolation Estimate = 16.4154; Entered P Value = 25; number of resampling = 80; Resamples Generated = 80; The Bootstrap Estimates Mean = 16.3993; standard deviation = 0.3723; lower confidence interval (95%) = 15.9225; upper confidence interval (95%) = 17.0320. resampling time in Seconds = 0.00; random Seed: -5333658.

**Table 19:** Chronic Toxicity of industrial bleaching effluent treated with ozone/UV (test 2).

Sample.	Number of Replicates	Concentration (%)	Mean response	Standard deviation	Pooled^a^ mean response
1	10	0	12.6	2.989	15.85
2	10	10	19.1	7.43	15.85
3	10	20	5.4	2.797	5.4
4	10	40	0	0	0

a Statistical validation. Linear Interpolation Estimate = 13.7919; Entered P Value = 25; number of resampling = 80; Resamples Generated = 80; The Bootstrap Estimates Mean = 13.8374; standard deviation = 0.5653; lower confidence interval (95%) = 13.2224; upper confidence interval (95%) = 14.6104. resampling time in Seconds = 0.00; random Seed: -119261604.

## 2 Experimental design, materials, and methods

The raw industrial wastewater was obtained from a *kraft* pulp mill with ECF sequence (Elemental Chlorine Free) located in Sao Paulo state – Brazil. Further information about the characteristics of this effluent can be found in Chaparro and Pires [3]. The pulp mill was treated biologically in a horizontal anaerobic immobilized biomass reactor (HAIB) for 306 days with an organic volumetric load of 2.33 kgCOD/m^3^. day and an hydraulic retention time of 25 h. The effluent from this reactor was subjected to ozone and ozone/UV oxidation tests without prior pH adjustment. The pH of the HAIB reactor effluent was close to 8.6. The samples were taken at regular intervals during the experimental period to evaluate the ecotoxicity and the genetic toxicity. Ecotoxicity assays were conducted according to the Brazilian standards [4,5], which were expressed in acute and chronic toxicity units using the linear interpolation method [6,7]. Table 2 shows a brief summary of the main requirements for the acute and chronic toxicity bioassays.

The genetic toxicity was performed according to the modified version of the *Grant`s protocol* [8]. The Genotoxicity index was evaluated based on the Chromosome aberrations (CA), Cytotoxicity was calculated through the mitotic index (MI) and the Mutagenic effect was assessed based on the micronucleus (MN) as follows:

(1) $$CA=\frac{number\;of\;cells\;with\;CA}{total\;number\;of\;\mathrm{observed}\;cells}\times \;100$$

(2) $$MI=\frac{number\;of\;dividing\;cells}{total\;number\;of\;\mathrm{observed}\;cells}\times 100$$

(3) $$MN=\frac{number\;of\;cells\;with\;MN}{total\;number\;of\;\mathrm{observed}\;cells}\times 10\text{0}$$

Finally, the statistical analysis considered the non-parametric *Kruskal–Wallis* applied by means of the BioEstat 5.0 software (https://bioestat.software.informer.com/5.0/).
